# Investigation of Ramped Compression Effect on the Dielectric Properties of Silicone Rubber Composites for the Coating of High-Voltage Insulation

**DOI:** 10.3390/ma15072343

**Published:** 2022-03-22

**Authors:** M. Hassan Raza, Safi Ullah Butt, Abraiz Khattak, Ahmad Aziz Alahmadi

**Affiliations:** 1 High Voltage Laboratory, Department of Electrical Power Engineering, U.S.-Pakistan Center for Advanced Studies in Energy, National University of Sciences and Technology (NUST), Islamabad 44000, Pakistan; mhassanraza423@gmail.com (M.H.R.); safibutt541@gmail.com (S.U.B.); 2 Department of Electrical Engineering, College of Engineering, Taif University KSA, P.O. Box 11099, Taif 21944, Saudi Arabia; aziz@tu.edu.sa

**Keywords:** silicone rubber, dielectric properties, compression, insulators, high voltage

## Abstract

The incorporation of inorganic oxide fillers imparts superior dielectric properties in silicone rubber for high-voltage insulation. However, the dielectric characteristics are influenced by the mechanical stress. The effects of ramped compression on the dielectric properties of neat silicone rubber (NSiR), 15% SiO_2_ microcomposite (SSMC), 15% alumina trihydrate (ATH) microcomposite (SAMC) and 10% ATH + 2% SiO_2_ hybrid composite (SMNC) are presented in this study. The dielectric constant and dissipation factor were measured before and after each compression especially in the frequency range of 50 kHz to 2MHz. Before the compression, SSMC expressed the highest dielectric constant of 4.44 followed by SMNC and SAMC. After the compression cycle, SAMC expressed a better dielectric behavior exhibiting dielectric constant of 7.19 and a dissipation factor of 0.01164. Overall, SAMC expressed better dielectric response before and after compression cycle with dielectric constant and dissipation factor in admissible ranges.

## 1. Introduction

Polymeric insulators offered numerous compensations as compared to the traditional insulation [1,2,3,4]. The advantages of polymeric insulation include higher resistance to flashovers, resistance to contaminations, better dielectric properties, higher tensile strength, ease of installation, less maintenance and lower cost [4,5,6]. Due to their superior properties, polymers find their usage as high voltage insulation and dielectric applications [7,8]. Dielectric properties are important as they act as stress relievers for the insulation system [9]. However, during service, the dielectric properties are influenced by the mechanical compression [10,11]. The recent developments in nanotechnology are assisting improvements in dielectric properties and mechanical endurance of polymeric insulation via the incorporation of inorganic oxide fillers into the polymer matrix [12,13].

The micro and nanocomposites of silicone rubber prepared with different inorganic oxide fillers such as titania (TiO_2_) and silica (SiO_2_) are reported to have improved dielectric properties as compared to neat silicone rubber [14,15]. In a study by Wang et al., it was found that with the increase in ZnO content in silicone rubber from 1% to 4%, the dielectric constant was increased [16]. Carpi et al. reported a dielectric constant of 8, accompanied by a dielectric loss of 0.9 in a titanium dioxide (TiO_2_)/silicone composite [17]. In another study, the effects of mechanical stresses on HTV silicone rubber were discussed and it was analyzed that all internal (residual) and external stresses had a great impact on the polymer material’s dielectric strength, electrical tree growth, space charge accumulation, and accumulated damage [18]. Zeng et al. discovered that with the inclusion of 50 wt.% nano TiO_2_ in silicone rubber, the dielectric constant was increased from 2.78 to 5.06 and the composites were thermally stable up to 400 °C [19].

Khattak et al. investigated the influence of silica addition and mechanical compression on the dielectric properties of epoxy and deduced that the composite with 5 wt.% addition of nano silica into epoxy had the best dielectric properties of all the composites, with an average dissipation factor (*DF*) of 0.09 and a dielectric constant of 6.23 [20]. For epoxy resin, mechanical stress altered the dielectric properties due to a change in tetragonal orthorhombic structures of epoxy into cubic structures [21].

Among the polymeric insulators, Room Temperature Vulcanized (RTV) silicone rubber is the best choice for the coating of high-voltage ceramic insulators [22,23]. The dielectric properties of silicone rubber are also influenced due to mechanical stresses. Therefore, it is important to study the effects of mechanical compression on the dielectric properties of RTV silicone composites.

In order to address the above-described significant gap, silica and alumina trihydrate filled micro, nano and hybrid composites are prepared. The effects of ramped compression with a ramp window of 3 MPa up to 12 MPa on the dielectric constant and dissipation factor are reported. The results of the dielectric constant and dissipation factor are compared before compression and after compression for the detailed analysis of the effects of ramped compression.

## 2. Procurement of Materials and Sample Fabrication

### 2.1. Procurement of the Materials

Room Temperature Vulcanized Silicone Rubber (RTV 615-A and 615-B), where RTV 615-A is the base and RTV-B is the curator, micro-silica (5 µm), micro-alumina tri-hydrate ATH (5 µm) and nano-silica (12 nm) were used in order to prepare the samples. Room Temperature Vulcanized silicone rubber was purchased from the German company Lanxess AG Chemicals. Silica-based fillers and ATH were procured from Degussa (Evonik) Chemical Co., Los Angeles, CA, USA Wuhan Newreach Chemical Co., Wuhan, China, respectively.

### 2.2. Preparation of Samples

**Figure 1:**
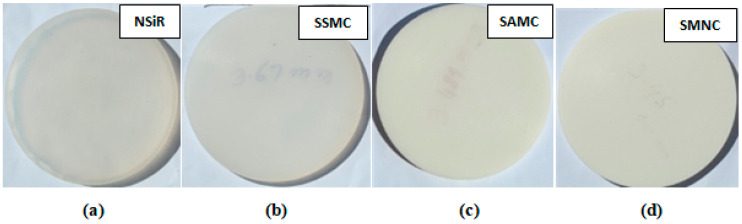
Images of the prepared samples (**a**) NSiR (**b**) SSMC (**c**) SAMC (**d**) SMNC.

The preparation of samples was conducted according to % weight of the filler and base polymer. Four different compositions, i.e., neat silicone rubber, 15% microsilica composite, 15% micro ATH composite and 10% ATH + 2% nanosilica composites were prepared. Table 1 shows the formulation summary and codes of the prepared composites. The base polymer and the curator were added with a ratio of 10:1 in all the samples. A high shear mixer and a sonicator were used for the preparation of samples. Prior to preparation, the fillers were placed in a vacuum oven for 16 h at 160 °C while RTV silicone rubber was vacuumed at 460 mm-Hg for a few hours. To obtain proper soaking of the dry fillers, RTV 615-A and dry fillers were mixed at a low speed of 3000 rpm in the first step. Following the soaking of the fillers, the mixture was mixed at a speed of 5000 rpm until no visible lumps remained in the mixture. To attain the maximum uniform dispersion, the addition of RTV 615-B in the mixture was carried out at low speed using the sonicator in the second step. For the purpose of degassing and de-bubbling, the mixture was vacuumed at 27 mm-Hg for few hours. The prepared slurry was then discharged in the molds having a diameter of 80 mm and a thickness of 3 mm in the third step. The post curing of the samples was performed at 90 °C for few hours in the last step. The images of the prepared samples are given in Figure 1.

## 3. Measurements and Methods

### 3.1. Measurement of Dielectric Properties

The dielectric properties were measured using the 7600 Plus LCR Meter of IET Labs USA. The samples were placed inside the inelastic dielectric cell LD-3 for the measurements. The diameter was 75 mm for all the samples so that they could fit in perfectly in the dielectric cell. The measurement range of frequency was set between 10 Hz–2 MHz. The range of 50 kHz- 2 MHz was chosen for the comparative analysis.

The fraction between the permittivity of material (*εm*) and that of free space (*εo*) is known as the dielectric constant (*κ*) as given in Equation (1):

(1) $$\kappa =\varepsilon_{\gamma }=\frac{\varepsilon m}{\varepsilon o}$$

The parallel capacitor capacitance is calculated by the formula given in Equation (2):

(2) $$C=\frac{A\varepsilon }{d}$$

where *A* is the area of the plates, *d* is the distance between the plates and *ε* is the permittivity of the material.

The percentage of uncertainty in measurement was 3%.

### 3.2. Conditions of Compression

**Figure 2:**
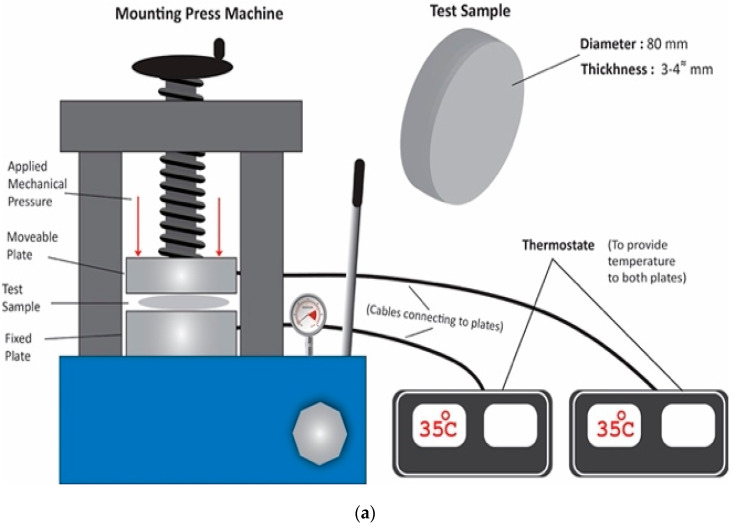

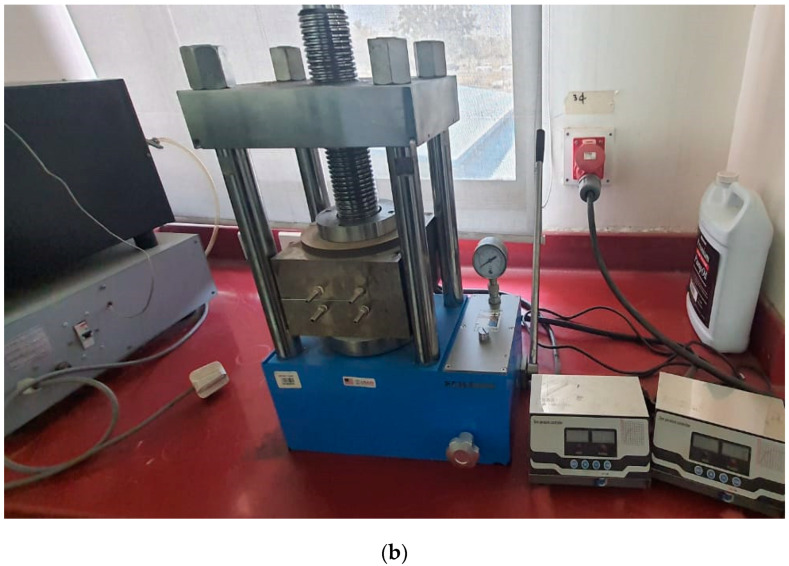
Compression setup (**a**) photo (**b**) schematic diagram.

A steel mold of 75 mm diameter was used to avoid the expansion of silicone rubber from the sides and ensure the uniform application of compression on the sample. The samples were placed inside the mold in order to avoid the expansion from the sides and to keep them contained, then the steel molds were placed in the hydraulic press. Spacing inside the steel mold was of the exact same diameter of the sample. The steel mold was designed in such a way to stop the expansion of the sample from the sides and allow compression in vertical form. The stress was increased from 0 MPa to the final value and the sample was subjected to the final value of compression value for 5 min. The compression stress was removed after 5 min and the samples were then taken out of the molds for dielectric properties testing in the dielectric analyzer. A China CY-600 compression setup was used for ramped compression. The schematic diagram and actual picture of the compression setup are shown in Figure 2. The samples were placed in the mold and heated at 35 °C. Ramped compression was applied with a ramp of 3 MPa up to 12 MPa. The dielectric properties were measured after each compression ramp.

## 4. Results and Discussions

### 4.1. Results at Low Frequencies

A practical capacitive system can be represented as an ideal capacitor with the incorporation of internal Equivalent Series Resistance (*ESR*) with the capacitor. In a dielectric medium, the existence of *ESR* indicates electrical conduction and dipole relaxation. The power loss in the capacitor is represented by dissipation factor (*DF*) and is given by the ratio of *ESR* and capacitive reactance.

(3) $$DF=\frac{ESR}{\left|X_{c}\right|}$$

For an AC source having frequency *f*, *X_c_* is given as:

(4) $$\left|X_{c}\right|=\frac{1}{2\pi fC_{m}}$$

Thus,

*DF* = *ESR*·(*2πfC_m_*) (5)

Using the Equations (3) and (4) we can deduce that

**Figure 3:**
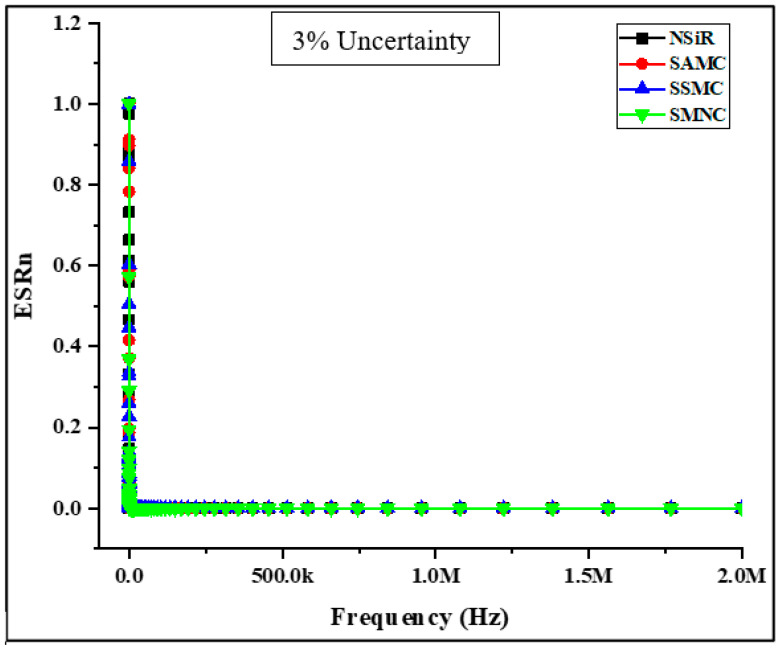
*ESR* values of samples against the frequency range.

*DF* = (*2πfESRCo*)·*K* (6)

where *k* is the dielectric constant and is given by *k* = *C_m_*/*C_o_*. The variation in dissipation factor is dependent on dielectric constant (*k*) if *ESR* of the material is known as given in Equation (6). According to Equation (6), the dissipation factor (*DF*) changes directly with the dielectric constant (*k*) if the *ESR* of the material is known. The higher the dielectric constant, the greater will be the ability of a material to store electric charge but for insulation purposes, low values of *DF* and dielectric constant (*k*) are desired. The measurement of *ESR* is quite tough due to high impedance and high displacement current at low frequencies and high frequencies, respectively. The impedance was between 100 Ω to 100 kΩ, and therefore, accurate measurements were expected at higher frequencies. At lower frequencies, the obtained results were misleading, as shown in Figure 3 where *ESRn* is the normalized value of *ESR* at a particular frequency. An abrupt increase in the values of *ESRn* was observed below 20 kHz frequency.

The increase in impedance beyond 100 kΩ may be the reason for this sudden increase. Due to the limitation of LCR meter in determining the accurate phase angle at such high impedance, the values of both *ESR* and *X*_c_ are not reliable, consequently making the measurements of *DF* faulty too. Due to the mentioned reasons, the measurements of *DF* should be considered in a frequency range where readings are reliable. Therefore, the range of 50 kHz to 2 MHz was considered for the accurate measurements.

### 4.2. Dielectric Constant

**Figure 4:**
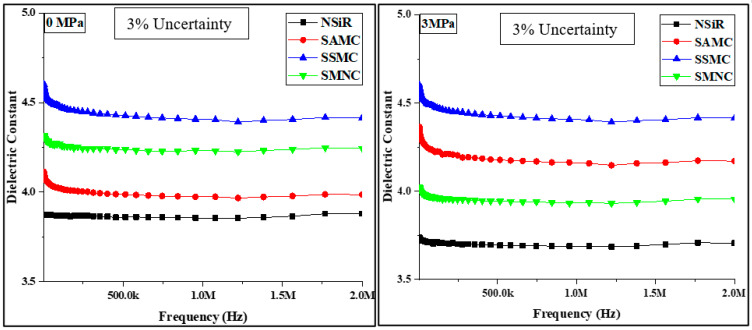

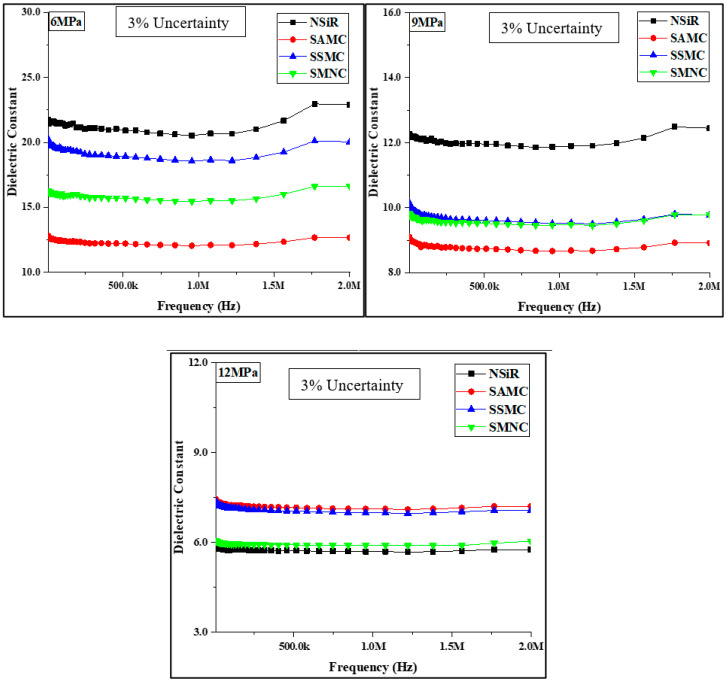
Results for dielectric constant of samples at different compressions.

The dielectric constant of all the samples were measured before compression and after each compression. The results for the dielectric constant of all the samples at 0 MPa, 3 MPa, 6 MPa, 9 MPa and 12 MPa are depicted in Figure 4. First, the samples were put one by one in LCR meter to find the results of dielectric constant and dissipation factor without compression. After that the samples were put in molds to heat them up to 35 °C then applied the compression for 3MPa. After that, when the molds containing samples were cooled down at room temperature, the samples were taken out. Successively used the LCR meter to find the dielectric constant and dissipation factor of each sample. The process was repeated for 0 MPa, 3 MPa, 6 MPa, 9 MPa and 12 MPa.

For all the samples, frequency dependency of the dielectric constant was observed. At lower frequencies, higher values of the dielectric constant were observed. However, at higher frequencies more stable values were recorded. This was due to the fact that at higher frequencies, dipoles are unable to align themselves against the applied field. Among the uncompressed samples, SSMC expressed the highest dielectric constant followed by SMNC and SAMC. Neat silicone rubber expressed the lowest dielectric constant at 0MPa. The higher dielectric constant of SSMC justifies the fact that the addition of silica can be more beneficial than ATH for the improvement of dielectric properties [24].

There was no significant change observed in the dielectric constant of all the samples at 3 MPa of compression. The dielectric constant of all the samples expressed a pronounced change in the values at 6 MPa of compression. For neat silicone rubber, the average value of the dielectric constant increased up to 21.24 from 3.7. Similarly, for SSMC, SAMC and SMNC, the average dielectric constant increased from 4.44 to 19.17, 4.2 to 12.3 and 3.95 to 15.84, respectively. This increase in dielectric constant can be attributed to the removal of voids and increased compactness after compression where the affect was more pronounced in neat silicone rubber [20,25]. A decrease in dielectric constant was seen for all the samples at 9 MPa. For neat silicone rubber, the average dielectric constant decreased from 21.24 to 12.03. Similarly, for SSMC, SAMC and SMNC, the average dielectric constant decreased from 19.17 to 9.67, 12.3 to 8.78 and 15.84 to 9.57, respectively. A further decrease in dielectric constant was seen at a compression of 12 MPa. Neat silicone rubber, SSMC, SAMC and SMNC expressed average dielectric constant of 5.73, 7.08, 7.19 and 5.93, respectively.

**Figure 5:**
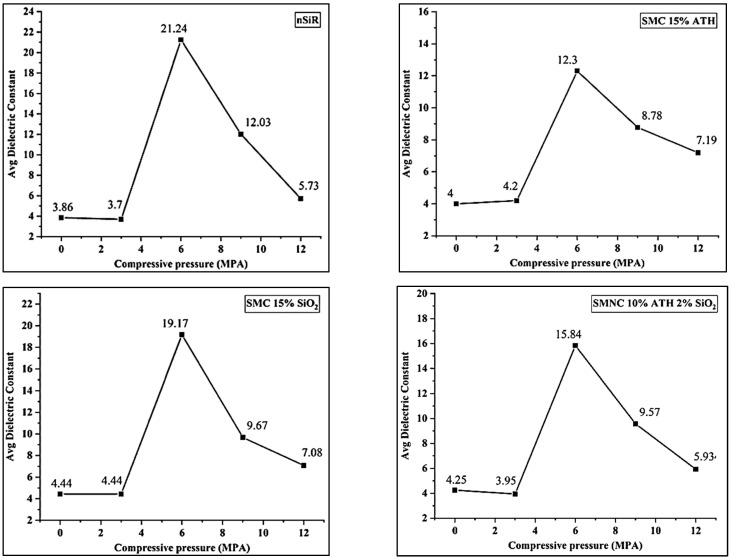
Average dielectric constant of samples at ramped compression.

The reduction in dielectric constant at 9 MPa and 12 MPa can be attributed to the destruction of the internal structure of the samples due to excessive compression [26,27]. The average values of dielectric constants of all the samples against the ramped compression are plotted in Figure 5.

### 4.3. Dissipation Factor

**Figure 6:**
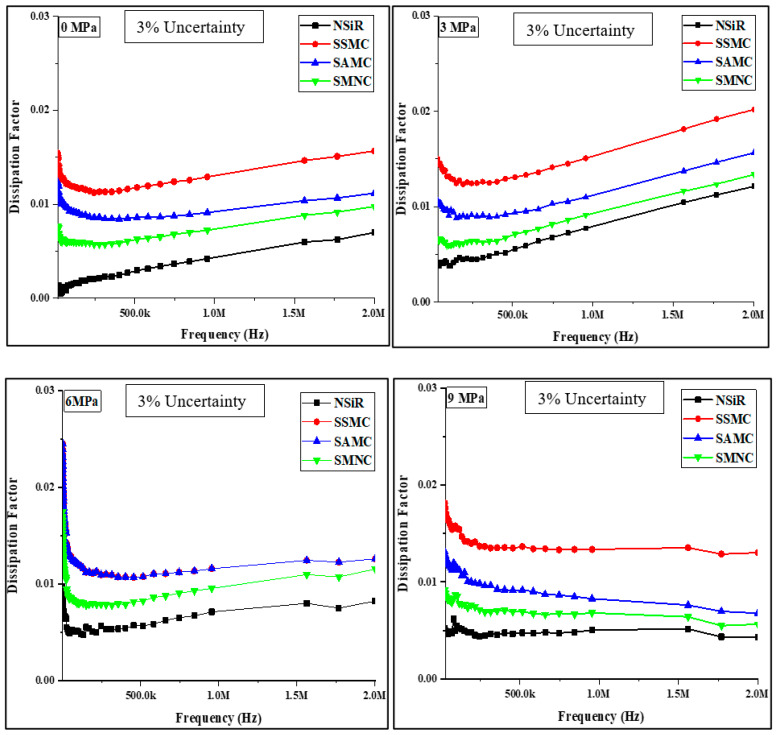

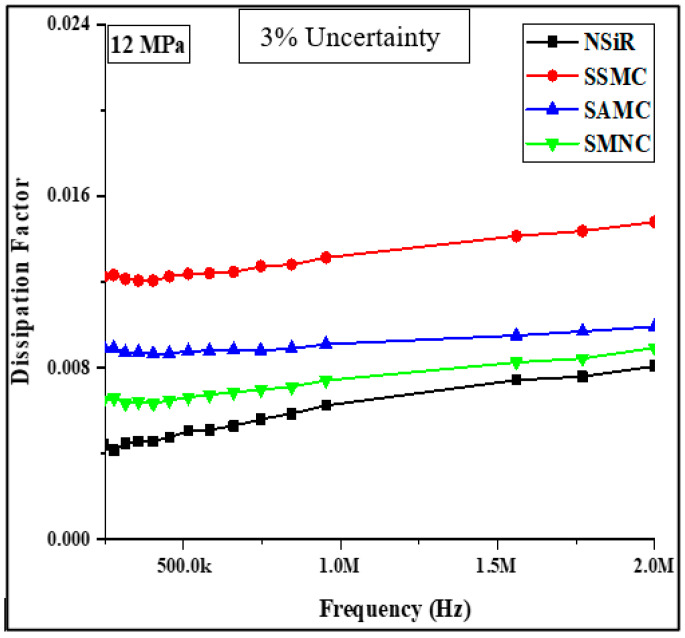
Results for dissipation factor of samples at different compressions.

**Figure 7:**
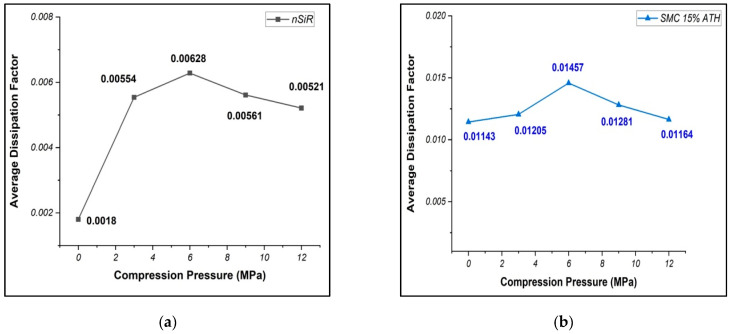

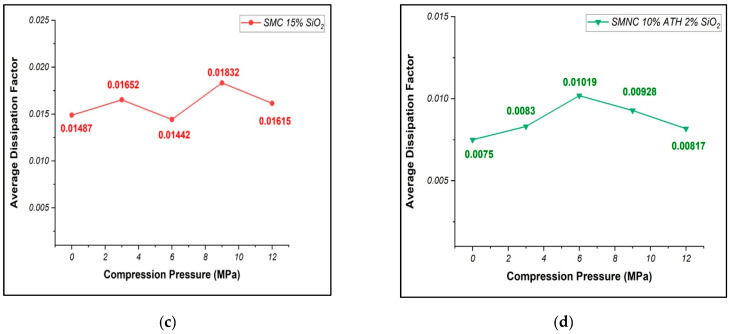
Average dissipation factor of samples (**a**) NSiR (**b**) SSMC (**c**) SAMC (**d**) SMNC.

The dissipation factor of all the samples was measured before compression at 0 MPa and after applying compressions of 3 MPa, 6 MPa, 9 MPa and 12 MPa against the frequency range depicted in Figure 6. While Average dissipation factors are given in Figure 7. For all the samples, frequency dependency of dissipation factor was observed. The dissipation factor followed the similar trend as of the dielectric constant. The higher values of dissipation factor were recorded at lower frequencies; however, at higher frequencies, more stable values were recorded. The dissipation factor was least for NSiR before compression with an average value of 0.0018 which increased 2.9 times after 5 compressions expressing final value of 0.00521. SAMC expressed an average value of 0.01143 for dissipation factor before compression. The final value of dissipation factor for SAMC was 0.01164 which is 1.02 times of 0.01143. The average value of dissipation factor for SSMC before compression was 0.01487 which increased up to 0.01615 after 5 compressions. The value of dissipation factor for SSMC was increased by 1.085 times.

Similarly, for SMNC, the dissipation factor increased by 1.09 times expressing an average value of 0.0075 and 0.00817 before and after 5 compressions, respectively. The trend in the average values of dissipation factor was the same as that of the dielectric constant. The highest dissipation factors were observed at 6 MPa compression for all the samples except SSMC which expressed the lowest dissipation factor at 6 MPa compression. The results of dielectric constant and dissipation factor of all the samples are compiled in Table 2.

### 4.4. Scientific Discussion

By resistance to the compression with respect to the dielectric properties four RTV based composite samples analyzed in this work can be arranged in the descending order as SAMC > SMNC > SSMC > NSiR.

The first and foremost thing found was the effect of compression on all samples i.e., Increase in dielectric constant was recorded up to 6 MPa and then decrease was recorded. This was due the facts all polymer exhibit reduction in voids and increase in compactness, extraction of trapped air that promoted solidified structure [28,29]. Secondly, the stress relaxation behavior of RTV silicone may have led to the enhancement due its high viscoelastic and stress relieving nature [30,31,32]. However, after the certain compression value (6 Mpa at 35 °C in this case), the effect was reversed which may be due to the elastic modulus limit [33]. The second important finding of this study was that for each composition dielectric behavior was not only different before compression but also after the compression neat silicone performed worst while ATH based composites performed best followed by silica-based composites. Improvement in dielectric properties of silica-based composites was due to the specific binding of silanol groups with the polymer network present on the surface of silica particles that significantly contributed to the overall structure stability against stresses [34,35]. The more pronounced effect in case of nano-silica-based composites was recorded due to the face that nano-silica has much larger specific area and holds almost 7.5 folds more silanol group on its surface than micro silica [36,37]. On the other hand, ATH-based micro composite having highest ATH loading performed best among all the samples (at 35 °C and 6 MPa). This can be due to the facts that ATH exhibit high temperature and pressure endurance properties and release its water content in high stressed application resulting in formation of aluminium oxide (Al_2_O_3_), high binding energy that lead to increase in heat [38] and mechanical stress [39] resistance of overall composite network.

## 5. Conclusions

Silicone rubber microcomposites and hybrid samples were prepared by adding fillers such Alumina tri-hydrate (ATH) and silica (SiO_2_). The mechanical compressions were performed on silicone rubber and its composites. The dielectric constant and average dissipation factors result were used to analyze the performance of samples under mechanical pressure. The results at initial frequencies were discarded due to machine limitations. Therefore, the results were compiled above 50 KHz. SSMC has shown the highest dielectric constant of 4.5 and dissipation factor 0.1487 before compression due to 15% percent of micro-silica loading. After compression at 3 MPa, there was an almost negligible effect on all the samples. However, after applying 6 MPa, the NSiR dielectric constant increased the most about 5.5 times. SAMC exhibited the least change in dielectric constant at 6 MPa and NSiR has showed the most change in its dielectric constant. For average dissipation factor, a similar trend was observed for NSiR and SAMC. Thus, in general electrical and high-voltage insulation as well as in the case of mechanically stressed application, ATH-based microcomposite with high concentration is performance is superior in order to have least effect on dielectric constant and dissipation factor. Keeping in view this finding further comparison with nano ATH-based composite at different concentration is recommended.

## Figures and Tables

**Table 1:** Sample formulations and codes.

Sr.	Sample Name	SiO_2_ Content (%wt.)	ATH Content (%wt.)	Sample Code
1	Neat Silicone Rubber	0%	0%	NSiR
2	Micro composite 1	15% µ SiO_2_	0%	SSMC
3	Micro composite 2	0%	15% µ ATH	SAMC
4	Nanocomposite	2% nano-SiO_2_	10% µ ATH	SMNC

**Table 2:** Comparison of dielectric constant and dissipation factor before and after compression.

Sr. #	Sample Name	Before Compression		After Compression		Effect of Ramped Compression
		Dielectric Constant	Dissipation Factor	Dielectric Constant	Dissipation Factor	
1	Neat Silicone Rubber	3.86	0.0018	10.6	0.00566	Most affected
2	Silicone Rubber Microcomposite 1	4.44	0.01487	10.9	0.01635	More affected
3	Silicone Rubber Microcomposite 2	4	0.01143	8.1	0.01277	Least affected
4	Silicone Rubber Hybrid Composite	4.2	0.0075	8.82	0.00899	Less affected

## Data Availability

The data presented in this study are available on request from the corresponding author.

## References

[1] Vlastos A., Gubanski S. (1991). Surface structural changes of naturally aged silicone and EPDM composite insulators. IEEE Trans. Power Deliv..

[2] Kim S., Cherney E., Hackam R. (1990). The loss and recovery of hydrophobicity of RTV silicone rubber insulator coatings. IEEE Trans. Power Deliv..

[3] Lee K.H., Kang M.S., Zhang S., Gu Y., Lodge T.P., Frisbie C.D. (2012). “Cut and stick” rubbery ion gels as high capacitance gate dielectrics. Adv. Mater..

[4] Amin M., Khattak A., Ali M. (2016). Life estimation and investigation of dielectric strength of multistressed high-voltage epoxy micro and nanocomposites. Micro Nano Lett..

[5] Gubanski S., Vlastos A. (1990). Wettability of naturally aged silicon and EPDM composite insulators. IEEE Trans. Power Deliv..

[6] Du B.X., Li A., Li J. (2016). Effects of AC and pulse voltage combination on surface charge accumulation and decay of epoxy resin. IEEE Trans. Dielectr. Electr. Insul..

[7] Bamji S., Bulinski A., Densley R., Chen Y. (1992). Threshold voltage for electrical tree inception in underground HV transmission cables. IEEE Trans. Electr. Insul..

[8] Afia R.S.A., Mustafa E., Tamus Z.A. Mechanical stresses on polymer insulating materials. Proceedings of the 2018 International Conference on Diagnostics in Electrical Engineering (Diagnostika).

[9] Cherney E. (2005). Silicone rubber dielectrics modified by inorganic fillers for outdoor high voltage insulation applications. IEEE Trans. Dielectr. Electr. Insul..

[10] Du B.X., Xu H., Li J. (2017). Effects of mechanical stretching on space charge behaviors of PP/POE blend for HVDC cables. IEEE Trans. Dielectr. Electr. Insul..

[11] da Silva R.F., Swinka Filho V. (2016). Analysis of electrical tracking by energy absorption during surface discharge in polymeric materials. IEEE Trans. Dielectr. Electr. Insul..

[12] Khattak A., Imran K., Faiza, Ali A., Ulasyar A., Haq A.U., Amin M., Khan A. (2020). Investigation of dielectric properties and methylene intactness under multiple environmental stresses for high voltage epoxy composites. Mater. Res. Express.

[13] Cho E., Chiu L., Lee M., Naila D., Sadanand S., Waldman S., Sussman D. (2021). Characterization of Mechanical and Dielectric Properties of Silicone Rubber. Polymers.

[14] Dang Z.M., Xia Y.J., Zha J.W., Yuan J.K., Bai J. (2011). Preparation and dielectric properties of surface modified TiO_2_/silicone rubber nanocomposites. Mater. Lett..

[15] Yang H., Gao Q., Xie Y., Chen Q., Ouyang C., Xu Y., Ji X. (2015). Effect of SiO_2_ and TiO_2_ nanoparticle on the properties of phenyl silicone rubber. J. Appl. Polym. Sci..

[16] Wang F.F., Yan D.D., Su Y., Lu Y.F., Xia X.F., Huang H.M. (2017). Research on the dielectric properties of nano-zno/silicone rubber composites. IOP Conference Series: Materials Science and Engineering.

[17] Carpi F., De Rossi D. (2005). Improvement of electromechanical actuating performances of a silicone dielectric elastomer by dispersion of titanium dioxide powder. IEEE Trans. Dielectr. Electr. Insul..

[18] Du B.X., Su J.G., Li J., Han T. (2017). Effects of mechanical stress on treeing growth characteristics in HTV silicone rubber. IEEE Trans. Dielectr. Electr. Insul..

[19] Zeng Y., Xiong C., Li J., Huang Z., Du G., Fan Z., Chen N. (2021). Structural, dielectric and mechanical behaviors of (La, Nb) Co-doped TiO_2_/Silicone rubber composites. Ceram. Int..

[20] Khattak A., Imran K., Ali A., Khan Z.S., Ulasyar A., Amin M., Khan A., Haq A.U. (2020). Effects of Compression and Silica Addition on the Dielectric Properties of Epoxy Composites. Arab. J. Sci. Eng..

[21] Girão H.T., Cornier T., Daniele S., Debord R., Caravaca M.A., Casali R.A., Mélinon P., Machon D. (2017). Pressure-Induced Disordering in SnO_2_ Nanoparticles. J. Phys. Chem. C.

[22] Raza M.H., Khattak A., Ali A., Butt S.U., Iqbal B., Ulasyar A., Alahmadi A.A., Ullah N., Khan A. (2021). Surface Recovery Investigation of Silicone Rubber Composites for Outdoor Electrical Insulation under Accelerated Temperature and Humidity. Polymers.

[23] Faiza F., Khattak A., Butt S., Imran K., Ulasyar A., Ali A., Khan Z., Mahmood A., Ullah N., Alahmadi A. (2021). Investigation of Hydrothermally Stressed Silicone Rubber/Silica Micro and Nanocomposite for the Coating High Voltage Insulation Applications. Materials.

[24] Meyer L.H., Cherney E.A., Jayaram S.H. (2004). The role of inorganic fillers in silicone rubber for outdoor insulation alumina tri-hydrate or silica. IEEE Electr. Insul. Mag..

[25] Yu J., Huo R., Wu C., Wu X., Wang G., Jiang P. (2012). Influence of interface structure on dielectric properties of epoxy/alumina nanocomposites. Macromol. Res..

[26] Luheng W., Tianhuai D., Peng W. (2007). Effects of conductive phase content on critical pressure of carbon black filled silicone rubber composite. Sens. Actuators A Phys..

[27] Wang L. (2016). Variations in the capacitance and dielectric constant of multi-wall carbon nanotube filled silicone rubber composite during compressive creep. Compos. Sci. Technol..

[28] Shivanand P., Sprockel O.L. (1992). Compaction behavior of cellulose polymers. Powder Technol..

[29] Naresh K., Khan K., Cantwell W., Umer R. (2022). Rate and temperature dependent compaction-creep-recovery and void analysis of compression molded prepregs. Compos. Part B Eng..

[30] Tobolsky A.V. (1956). Stress Relaxation Studies of the Viscoelastic Properties of Polymers. J. Appl. Phys..

[31] Stein J., Prutzman L.C. (1988). Stress relaxation studies of model silicone RTV networks. J. Appl. Polym. Sci..

[32] Michel S., Zhang X.Q., Wissler M., Löwe C., Kovacs G. (2009). A comparison between silicone and acrylic elastomers as dielectric materials in electroactive polymer actuators. Polym. Int..

[33] Koseki K., Arita T., Tabata K., Nohara T., Sato R., Nagano S., Masuhara A. (2021). Effect of Surface Silanol Density on the Proton Conductivity of Polymer-Surface-Functionalized Silica Nanoparticles. ACS Sustain. Chem. Eng..

[34] Okel T.A., Waddell W.H. (1995). Effect of Precipitated Silica Physical Properties on Silicone Rubber Performance. Rubber Chem. Technol..

[35] Zhang C., Liu L., Zhang Z., Pal K., Kim J.K. (2011). Effect of Silica and Silicone Oil on the Mechanical and Thermal Properties of Silicone Rubber. J. Macromol. Sci. Part B.

[36] Khattak A., Amin M., Iqbal M., Abbas N. (2018). Life estimation and analysis of dielectric strength, hydrocarbon backbone and oxidation of high voltage multi stressed EPDM composites. Mater. Res. Express.

[37] Khattak A., Amin M., Iqbal M. (2018). Long term accelerated aging investigation of an epoxy/silica nanocomposite for high voltage insulation. J. Polym. Eng..

[38] Zolriasatein A., Navazani S., Abadchi M.R., Noori N.R. (2021). Two-component room temperature vulcanized silicone-rubber (RTV2) properties modification: Effect of aluminum three hydrate and nanosilica additions on the microstructure, electrical, and mechanical properties. J. Mater. Sci. Mater. Electron..

[39] Petersen M.R., Chen A., Roll M., Jung S., Yossef M. (2015). Mechanical properties of fire-retardant glass fiber-reinforced polymer materials with alumina tri-hydrate filler. Compos. Part B Eng..

